# Infestation and Distribution Patterns of Ectoparasites on the Red-Eared Harvest Mouse (*Micromys erythrotis*) in Southwest China

**DOI:** 10.3390/vetsci13101011

**Published:** 2026-09-24

**Authors:** Jin-Xuan Chen, Wen-Yu Song, Peng-Wu Yin, Yan Lv, Pei-Ying Peng, Xian-Guo Guo

**Affiliations:** 1 Institute of Pathogens and Vectors, Key Laboratory of Ectoparasite Systematics and Evolution of Yunnan Provincial Education Department, Yunnan Provincial Key Laboratory for Zoonosis Control and Prevention, Dali University, Dali 671000, China; 2 Institute of Microbiology, Qujing Health Medical College, Qujing 655100, China

**Keywords:** *Micromys erythrotis*, rodents, ectoparasites, fleas, sucking lice, ticks, mites

## Abstract

Given the paucity of research on the red-eared harvest mouse (*Micromys erythrotis*) and its associated ectoparasites, this study aims to illustrate the infestation and distribution patterns of ectoparasites on this mouse in southwest China. A retrospective analysis was conducted based on field investigations and taxonomic identification across five provinces in southwest China between 2000 and 2024. Infestation indices, spatial distribution indices, and generalized linear mixed models (GLMMs) were used for analysis. From the 273 *M. erythrotis* captured, 1349 ectoparasites were identified comprising 54 species, including nine species of vectors or potential vectors in the transmission of specific zoonotic pathogens. The overall infestation indices were *P_M_* = 66.30%, *MA* = 4.94, and *MI* = 7.45. Sucking lice accounted for the highest number of parasite individuals (*C_r_* = 45.59%), whereas gamasid mites exhibited the highest species richness (*S* = 24). All spatial distribution indices exceeded the critical values for an aggregated distribution. Adult hosts exhibited higher infestation than juveniles. Co-infestation with two or more parasite groups was observed in 29.30% of *M. erythrotis* individuals. In conclusion, *M. erythrotis* individuals were infested with diverse species of ectoparasites, dominated by sucking lice and gamasid mites. The coexistence of multiple vector species may have potential significance in epidemiology.

## Simple Summary

Wild rodents often carry tiny parasites on their skin, which can sometimes spread diseases to humans. However, very little is known about the parasites living on the red-eared harvest mouse, a small wild rodent found in southwest China. This study aimed to identify the types and distribution of these parasites. By examining mice collected across five provinces from 2000 to 2024, researchers found that this mouse harbored a diverse species of ectoparasites. Out of 273 mice examined, over 66 percent carried parasites, yielding a total of 1349 individual parasites. These included fleas, sucking lice, mites, and ticks, with sucking lice displaying the greatest abundance of individuals and the greatest variety of species being observed for gamasid mites. Many mice were infested with multiple types of parasites simultaneously. Notably, nine species of ectoparasites can act as the vectors or potential vectors in the transmission of specific zoonotic diseases. The findings provide scientific information on ectoparasites, which would be beneficial to future monitoring and risk assessment of some zoonotic diseases and related vectors in southwest China.

## 1. Introduction

Rodents (rats, mice, voles, etc.) act not only as major agricultural and forestry pests but also as critical reservoir hosts and sources of infection for numerous zoonotic pathogens [1]. Ectoparasites on rodents often involve five groups of arthropods, namely fleas, sucking lice, gamasid mites, chiggers, and ticks. Some ectoparasites on rodents can serve as vectors of some zoonotic diseases (zoonoses). For instance, fleas are the vector of plague and murine typhus. Chiggers are the exclusive vector of scrub typhus. Ticks transmit a number of tick-borne zoonoses such as tick-borne encephalitis and Lyme disease [2,3,4]. 

The red-eared harvest mouse (*Micromys erythrotis*) is a small-sized rodent (usually 4.8 to 7.5 g in body weight and 51 to 65 mm in body length), belonging to the genus Micromys, family Muridae, and order Rodentia. Being a typical wild-dwelling rodent, *M. erythrotis* is often found in various wild habitats of southern and southwestern China [5]. To date, the limited reports on *M. erythrotis* have primarily focused on its taxonomic status, geographical distribution, and phylogeny, without specific studies on its ectoparasites. Based on field surveys and taxonomic identification across the five provincial regions of southwest China between 2000 and 2024, the present study reports, for the first time, the infestation and distribution patterns of ectoparasites on *M. erythrotis* in the region, aiming to enrich the knowledge of this special mouse and its associated ectoparasites.

## 2. Materials and Methods

### 2.1. Field Sampling and Host Collection

Field investigations were previously conducted at 117 survey sites across the five provincial regions of southwest China (Yunnan, Guizhou, Sichuan, Chongqing, and Xizang) between 2000 and 2024 (Figure 1). The field surveys were mainly conducted from March to May in spring and from September to November in autumn. The red-eared harvest mouse (*M. erythrotis*) and other rodent hosts were captured alive using mousetraps (18 × 12 × 9 cm, Guixi Mousetrap Apparatus Factory, Guixi, Jiangxi, China) baited with peanuts, corns, or other food baits. In different habitats, mousetraps were placed in the late afternoon or evening and checked the following morning. The surveyed habitats involved indoor habitats (houses, stables, barns, and nearby surroundings) and outdoor habitats (farmlands, bushes, and woodlands). In dry areas, mousetraps were arranged in straight lines with a row spacing of 20 m and adjacent traps spaced 5 m apart. The traps were flexibly placed along the base of walls indoors and along the banks of paddy fields. At each survey site, approximately 200 mousetraps were placed in different habitats every day for about 15 days, with about 3000 traps per site in total [6]. The captured rodents were identified at the species level based on morphological characteristics and body measurements [7,8]. Based on the taxonomic identification of rodent hosts, the red-eared harvest mouse (*M. erythrotis*) was chosen as the host target of the present study. The capture and use of rodents were approved by local wildlife authorities and the Animals’ Ethics Committees of Dali Medical College (1990–2000) and Dali University (after 2001) with approval codes DLYXY1990-0109, DLXY2001-1116, and DLDXLL2020-1104.

### 2.2. Ectoparasite Collection and Identification

Ectoparasites (fleas, sucking lice, gamasid mites, chiggers, and ticks) on each rodent host were routinely collected and preserved in 70% or 75% ethanol. After being rinsed in distilled water, gamasid mites and chiggers were directly mounted onto glass slides with Hoyer’s medium. Fleas, sucking lice and ticks were first digested in 5–10% sodium hydroxide (or potassium hydroxide) solution. After being rinsed in distilled water, the digested arthropods (fleas, sucking lice and ticks) were successively dehydrated using different concentrations of ethanol (30%, 50%, 70%, 80%, 90%, 95%, and 100%). The dehydrated specimens were cleared with xylene and then mounted onto glass slides with Canadian balsam or firglue [6]. After being dried in an oven, the mounted specimens were taxonomically identified to species under a microscope (Olympus Corporation, Tokyo, Japan) [9,10,11,12,13].

### 2.3. Analysis on Parasite Infestation

The constituent ratio (*C_r_*) was used to describe the proportion of ectoparasite individuals, whereas species richness (*S*) was used to describe the number of ectoparasite species. Prevalence (*P_M_*) was defined as the percentage of examined hosts infested with ectoparasites, mean abundance (*MA*) was defined as the mean number of ectoparasites per examined host, and mean intensity (*MI*) was defined as the mean number of ectoparasites per infested host [14]. Differences in *P_M_* between host sex and age groups were assessed using Pearson’s chi-square test (or Fisher’s exact test when appropriate), whereas differences in *MA* and *MI* were assessed using the Mann–Whitney U test. As *M. erythrotis* is a small-bodied murid rodent, the Meeh–Rubner equation previously evaluated in mice was considered more appropriate for estimating its body surface area in the absence of a species-specific equation. The body surface area was estimated as follows:

Body surface area (cm^2^) = 9.82 × W^(2/3)^,

where W is body weight (g). The infestation burden of ectoparasites per unit of host body was calculated based on the measurement of *M. erythrotis*’ body weight, body length, and body surface area [15,16].

### 2.4. Spatial Distribution Analysis

The dispersion coefficient (*C*), clumping index (*I*), Cassie’s index (*C_A_*), and patchiness index (m*/*m*) were used to analyze the spatial distribution patterns of ectoparasites among different individuals of *M. erythrotis* hosts [14]. These indices were calculated for the total ectoparasite burden and separately for each ectoparasite group (fleas, sucking lice, gamasid mites, chiggers, and ticks). All four indices were retained as complementary measures of aggregation.

### 2.5. GLMM Analysis

Generalized linear mixed models (GLMMs) were used to analyze the effects of host and environmental factors on ectoparasite infestation. For the prevalence model, host infestation status (infested or uninfested) was used as the response variable, with a binomial distribution and a logit link function. For the parasite count model, the total number of ectoparasites on each host was used as the response variable, with a negative binomial distribution (nbinom2) and a log link function. Host sex, age, body weight, body length, habitat type, landscape type, and elevation group were included as fixed effects. These predictors were specified a priori based on the study objectives and their biological relevance. The sampling site was included as a random intercept to account for clustering among hosts collected from the same sampling sites. The GLMM analyses were performed in R version 4.5.3 using the glmmTMB package (version 1.1.14). The significance of fixed effects was evaluated using Wald chi-square tests, and model estimates were reported as odds ratios (ORs) for the prevalence model and incidence rate ratios (IRRs) for the parasite count model, together with 95% confidence intervals. Model diagnostics were performed using simulated residuals, including tests for dispersion and zero inflation. Potential multicollinearity among the fixed-effect predictors was assessed using variance inflation factors (VIFs) [17]. Spearman’s rank correlation coefficient (*ρ*) was used to assess pairwise relationships among the five ectoparasite groups, considering the non-normal distribution and zero values in the ectoparasite count data. A *p*-value < 0.05 was considered statistically significant. The relevant calculation formulas can be found in the relevant literature [14,15,16,17].

## 3. Results

### 3.1. Species Composition and Overall Infestation of Ectoparasites on Micromys erythrotis

A total of 273 red-eared harvest mice (*M. erythrotis*) were captured at 25 of 117 survey sites in southwest China (Figure 1). From their body surfaces, 1349 ectoparasites were collected and identified as belonging to 5 orders, 13 families, 25 genera, and 54 species in five groups: fleas, sucking lice, gamasid mites, chigger mites, and ticks. Among the 54 ectoparasite species, nine species have been documented as vectors or potential vectors of specific zoonotic pathogens (Table 1). The overall infestation indices of ectoparasites were *P_M_* = 66.30% (181/273), *MA* = 4.94 parasites/per host, and *MI* = 7.45 parasites/per infested host. Among the five parasite groups, sucking lice accounted for 45.59% of the 1349 ectoparasites, representing the highest constituent ratio (*C_r_* = 45.59%, 615/1349), whereas gamasid mites had the highest species richness (*S = 24*), with relatively high infestation indices (Table 2). Of the seven flea species, *Neopsylla specialis* was dominant (*C_r_* = 90.24%, 74/82). Of the two sucking louse species, *Hoplopleura longula* was slightly more abundant (*C_r_* = 54.63%, 336/615) than *Polyplax gracilis* (*C_r_* = 45.37%, 279/615). Of the 24 gamasid mite species, *Laelaps spatuliformis* was dominant (*C_r_* = 44.53%, 175/393). Of the 17 chigger mite species, *Leptotrombidium jishouense* was dominant (*C_r_* = 35.78%, 83/232). Of the four tick species, *Rhipicephalus haemaphysaloides* was dominant (*C_r_* = 74.07%, 20/27). The nine species with documented vector roles (or potential vector roles) were *Neopsylla specialis*, *Leptopsylla segnis*, *Stenischia humilis, Haemolaelaps glasgowi*, *Leptotrombidium scutellare*, *Leptotrombidium jishouense*, *Rhipicephalus haemaphysaloides*, *Ixodes granulatus,* and *Haemaphysalis doenitzi* (Table 1). For the five groups of ectoparasites (fleas, sucking lice, chiggers, gamasid mites, and ticks together), the spatial distribution indices exceeded the threshold values for an aggregated distribution (*C* > 1, *I* > 0, *CA* > 0, and m*/*m* > 1), with *C* = 13.75, *I* = 12.75, *CA* = 2.58, and m*/*m* = 3.58. When each group of parasites was analyzed separately, all five ectoparasite groups also showed aggregated distributions, with the corresponding spatial distribution indices presented in Table 2.

**Table 1:** Taxonomic identification of ectoparasites on *Micromys erythrotis* in southwest China (2000–2024).

Taxonomic Taxa of Ectoparasites	Collected Species and Individuals
Fleas	Total fleas: 82 individuals, 7 species, 7 genera, 5 families
Hystrichopsyllidae	*Neopsylla specialis* * (74); *Aviostivalius klossi bispiniformis* (2); *Lentistivalius affinis* (1)
Leptopsyllidae	*Leptopsylla segnis* * (2)
Ctenophthalmidae	*Ctenophthalmus yunnanus* (1)
Pygiopsyllidae	*Stenischia humilis* * (1)
Ceratophyllidae	*Frontopsylla spadix* (1)
Sucking lice	Total sucking lice: 615 individuals, 2 species, 2 genera, 2 families
Hoplopleuridae	*Hoplopleura longula* (336)
Polyplacidae	*Polyplax gracilis* (279)
Gamasid mites	Total gamasid mites: 393 individuals, 24 species, 9 genera, 4 families
Laelapidae	*Laelaps spatuliformis* (175); *Dipolaelaps anourosorecis* (65); *Haemolaelaps glasgowi* * (28); *Laelaps nuttalli* (27); *Laelaps micromydis* (20); *Laelaps turkestanicus* (14); *Laelaps xingyiensis* (13); *Laelaps fukienensis* (7); *Laelaps echidninus* (6); *Laelaps guizhouensis* (6); *Laelaps chini* (5); *Hypoaspis ovatus* (2); *Proctolaelaps pygmaeus* (2); *Laelaps hongaiensis* (2); *Laelaps algericus* (2); *Hypoaspis pavlovskii* (1); *Laelaps jettmari* (1); *Laelaps agilis* (1); *Tricholaelaps myonysognathus* (1); *Gymnolaelaps sinensis* (1)
Hirstionyssidae	*Hirstionyssus sunci* (8)
Ascidae	*Lasioseius medius* (3); *Lasioseius praevius* (1)
Haemogamasidae	*Haemogamasus monticola* (2)
Chigger mites	Total chigger mites: 232 individuals, 17 species, 4 genera, 1 family
Trombiculidae	*Leptotrombidium jishouense* * (83); *Leptotrombidium fujianense* (67); *Leptotrombidium hiemale* (20); *Gahrliepia eurypunctata* (12); *Leptotrombidium wangi* (9); *Leptotrombidium xiaguanense* (7); *Leptotrombidium rusticum* (6); *Huabangsha megachela* (6); *Leptotrombidium gongshanense* (4); *Gahrliepia yunnanensis* (4); *Leptotrombidium sinicum* (3); *Leptotrombidium eothenomydis* (3); *Leptotrombidium xiaowei* (3); *Leptotrombidium scutellare* * (2); *Walchia micropelta* (1); *Gahrliepia longipedalis* (1); *Gahrliepia linguipelta* (1)
Ticks	Total ticks: 27 individuals, 4 species, 3 genera, 1 family
Ixodidae	*Rhipicephalus haemaphysaloides* * (20); *Ixodes granulatus* * (3); *Haemaphysalis doenitzi* * (3); *Haemaphysalis canestrinii* (1)
Total ectoparasites	1349 individuals, 54 species, 25 genera, 13 families, 5 orders

Note: The numbers in parentheses are the individuals of each ectoparasite species. * Species marked with an asterisk have been documented as vectors or potential vectors of relevant zoonotic diseases [3,18,19,20,21,22,23,24].

### 3.2. Effects of Host and Environmental Factors on Ectoparasite Infestation

According to the recorded body weight and body length of each mouse host (*M. erythrotis*), the average body weight and body length of *M. erythrotis* were calculated as 8.79 ± 2.05 g/per host and 67.55 ± 6.13 mm/per host. Based on the Meeh–Rubner formula, the mean body surface area was estimated as 41.57 ± 6.43 cm^2^. The ectoparasite burdens per unit of *M. erythrotis* were then calculated as follows: 0.69 ± 0.91 parasites/g for body weight, 0.85 ± 1.15 parasites/cm for body length, and 0.14 ± 0.18 parasites/cm^2^ for body surface area.

Of the 273 *M. erythrotis* individuals captured, 266 mice (139 males and 127 females) had sex records and 272 mice had age records (255 adults and 17 juveniles). Based on recorded data on hosts’ sexes and ages, infestation indices for ectoparasites were calculated for different sexes and ages of *M. erythrotis*. The infestation indices of ectoparasites for male *M. erythrotis* (*P_M_* = 68.35%, *MA* = 5.13, *MI* = 7.51) were slightly higher than those for the females (*P_M_* = 65.35%, *MA* = 4.57, *MI* = 7.00), but without statistical significance (*p* > 0.05). The adult hosts (*M. erythrotis*) exhibited higher infestation (*P_M_* = 67.06%, *MA* = 5.21, *MI* = 7.77) than the juveniles (*P_M_* = 52.94%, *MA* = 1.18, *MI* = 2.22), with statistical significance for *MA* and *MI* (*p* < 0.05) but without statistical significance for *P_M_* (*p* > 0.05) (Figure 2). The generalized linear mixed models (GLMMs) for infestation indices (prevalence and parasite count) were established to analyze the effects of host and environmental factors on ectoparasite infestation. The models incorporated host characteristics (sex, age, body weight, and body length), habitats (agricultural and non-agricultural areas), geographical landscapes (basins and mountainous areas), and altitude gradients (<1000 m, 1000–2000 m, and >2000 m) as variables. The analysis revealed no statistically significant associations between these host and environmental factors and prevalence or parasite count (Table 3, *p* > 0.05). Collinearity diagnostics showed VIF values ranging from 1.02 to 3.36 for the prevalence model and from 1.04 to 1.92 for the parasite count model, indicating no substantial multicollinearity among the included predictors.

### 3.3. Co-Infestations and Relationships Among Different Parasite Groups

The Sankey diagram in Figure 3a visualized the proportions (%) of hosts with single-group infestation and co-infestations among different parasite groups (fleas, sucking lice, gamasid mites, chiggers, and ticks) on *M. erythrotis*. Of the 273 *M. erythrotis* hosts, 101 mouse hosts were only infested with a single parasite group (37.00%, 101/273), and 80 mouse hosts were co-infested with two or more parasite groups (29.30%, 80/273). The remaining 92 hosts were negative for ectoparasite infestation (33.70%, 92/273). The mouse hosts co-infested with sucking lice and gamasid mites accounted for 40.00% of total 80 co-infested hosts (40%, 32/80), and those co-infested with sucking lice and fleas came next (17.50%, 14/80) (Figure 3a). Spearman’s rank correlation analysis revealed that slight positive and negative correlations existed among five parasite groups with low values of correlation coefficients. For instance, a weak positive correlation was found between sucking lice and gamasid mites (*ρ* = 0.1928, *p* < 0.01) and between sucking lice and fleas (*ρ* = 0.2703, *p* < 0.001) (Figure 3b).

## 4. Discussion

The present study systematically reported the infestation and distribution patterns of ectoparasites on the mouse *M. erythrotis* in southwest China for the first time. Although some previous reports on ectoparasitic mites involved some fragmentary information regarding *M. erythrotis* and its ectoparasites, they did not completely explore parasite infestation and distribution for this mouse [25,26]. For instance, a previous study on the gamasid mite *Laelaps echidninus* showed that *L. echidninus* could infest *M. erythrotis* in southwest China [25], which is consistent with the present study. A local survey on chigger mites in northern Yunnan of southwest China recorded only one *M. erythrotis*, on which no chiggers were found [26]. In contrast, the present study covered five groups of ectoparasites on *M. erythrotis*, in which 54 parasite species were found, indicating that *M. erythrotis* can harbor diverse species of ectoparasites. The ectoparasite community on *M. erythrotis* showed clear differences among five ectoparasite groups, namely fleas, sucking lice, gamasid mites, chiggers, and ticks. Of the five parasite groups, sucking lice were numerically dominant, accounting for 45.59% (615/1349) of all ectoparasite individuals, whereas gamasid mites exhibited the highest species richness (*S* = 24). The infestation indices (*P_M_*, *MA* and *MI*) reflect the infestation status of *M. erythrotis* with five parasite groups. The ectoparasite burden standardized by host body surface area was 0.14 ± 0.18 parasites/cm^2^, providing an additional measure of parasite load relative to host body dimensions.

Southwest China is an important focus of many zoonoses, including plague, murine typhus, scrub typhus, hemorrhagic fever with renal syndrome (HFRS), tick-borne encephalitis, rickettsioses, Lyme disease, and babesiosis [20,21,22,23,24,27]. Of the nine species with documented vector roles or potential vector roles in the present study, three were flea species. The fleas *Neopsylla specialis* and *Leptopsylla segnis* have been proven to be confirmed vectors of plague, and *L. segnis* is considered a secondary vector with relatively low vector efficiency [18,19]. Besides the two vector flea species, *Stenischia humilis* has been reported from plague foci in Yunnan Province, and *Yersinia pestis* has been isolated from this flea species multiple times [20]. Two chigger species (*L. scutellare* and *L. jishouense*) are confirmed vectors of scrub typhus in China [3]. The gamasid mite *H. glasgowi* can act as a potential vector for some zoonoses such as HFRS and tick-borne encephalitis [21]. The tick *I. granulatus* is an important vector of Lyme disease, *R. haemaphysaloides* has been documented as a potential vector of human babesiosis, and *H. doenitzi* has been reported to be capable of transmitting rickettsiae and *Borrelia* (Table 1) [22,23,24]. The occurrence of these nine species with documented vector roles or potential vector roles on *M. erythrotis* highlights the need for further investigation of their potential epidemiological significance.

The infestation indices of ectoparasites for male hosts were slightly higher than those for the females, and adult hosts exhibited higher infestation than the juveniles (Figure 2). This may reflect the sex and age biases of hosts to ectoparasite infestation. Some previous studies have indicated that male animals (including rodents) often consume more energy than female ones because of mating competition, and the high level of testosterone in male hosts can compromise their immune resistance and make them more susceptible to parasite infestations [28]. Compared to juveniles, adult rodents possess a large body surface area and they often have a wide range of activities due to foraging needs, which increases their probability of exposure to parasites, including ectoparasites [29]. The infestation of ectoparasites may be influenced by a variety of factors, including host factors and environmental factors [17]. In the present study, however, the GLMM analysis did not detect statistically significant associations between the examined host or environmental factors and either ectoparasite prevalence or parasite count (Table 3, *p* > 0.05). Although adults showed significantly higher *MA* and *MI* than juveniles in the univariate analysis, host age was not significantly associated with parasite count in the GLMM. This suggests that the significant age-group difference observed in the univariate analysis may not be attributable to age alone, as it did not remain statistically significant when other host and environmental factors were considered simultaneously in the GLMM. In addition, the highly unbalanced sample size between adults and juveniles (255 vs. 17) may have reduced the statistical power for detecting age-related effects and affected the reliability of comparisons between the two age groups. Based on the current results, we cannot determine how host and environmental factors influence the infestation of ectoparasites on *M. erythrotis*, and therefore more studies are still needed in the future.

Co-infestation with two or more parasite groups was observed in 80 of the *M. erythrotis* individuals (29.30%, 80/273). The results of the present study are consistent with some previous reports [30], implying that *M. erythrotis* is frequently co-infested by multiple ectoparasite groups. Although there was co-infestation with multiple parasites on *M. erythrotis*, only weak positive and negative correlations were observed among the five parasite groups, with correlation coefficients (*ρ*) close to 0 (Figure 3b). This result may imply that the parasitism of five parasite groups may happen independently on the body surface of *M. erythrotis*, without mutual attraction or repulsion. In addition, pairwise Spearman correlations may not fully characterize the co-occurrence patterns among different ectoparasite groups, with many zero counts in the original data of the present study.

Although the present study systematically revealed the infestation and distribution patterns of ectoparasites on *M. erythrotis* in southwest China, there are still some limitations in this study. In addition to the relatively few individuals found for the parasite species with documented vector roles or potential vector roles, no pathogen detection was conducted for these parasite species in the present study. From the results of the present study, we cannot evaluate the potential risk of these vector species in the transmission of relevant zoonotic diseases in southwest China, and more research incorporating pathogen detection is still needed in the future. Another limitation is that, because this retrospective study was based on historical field records, host age was recorded only as adult or juvenile, and detailed reproductive-status data, particularly for female hosts, were unavailable. Future studies should use standardized age criteria and record reproductive status to better assess their effects on ectoparasite infestation.

## Figures and Tables

**Figure 1:**
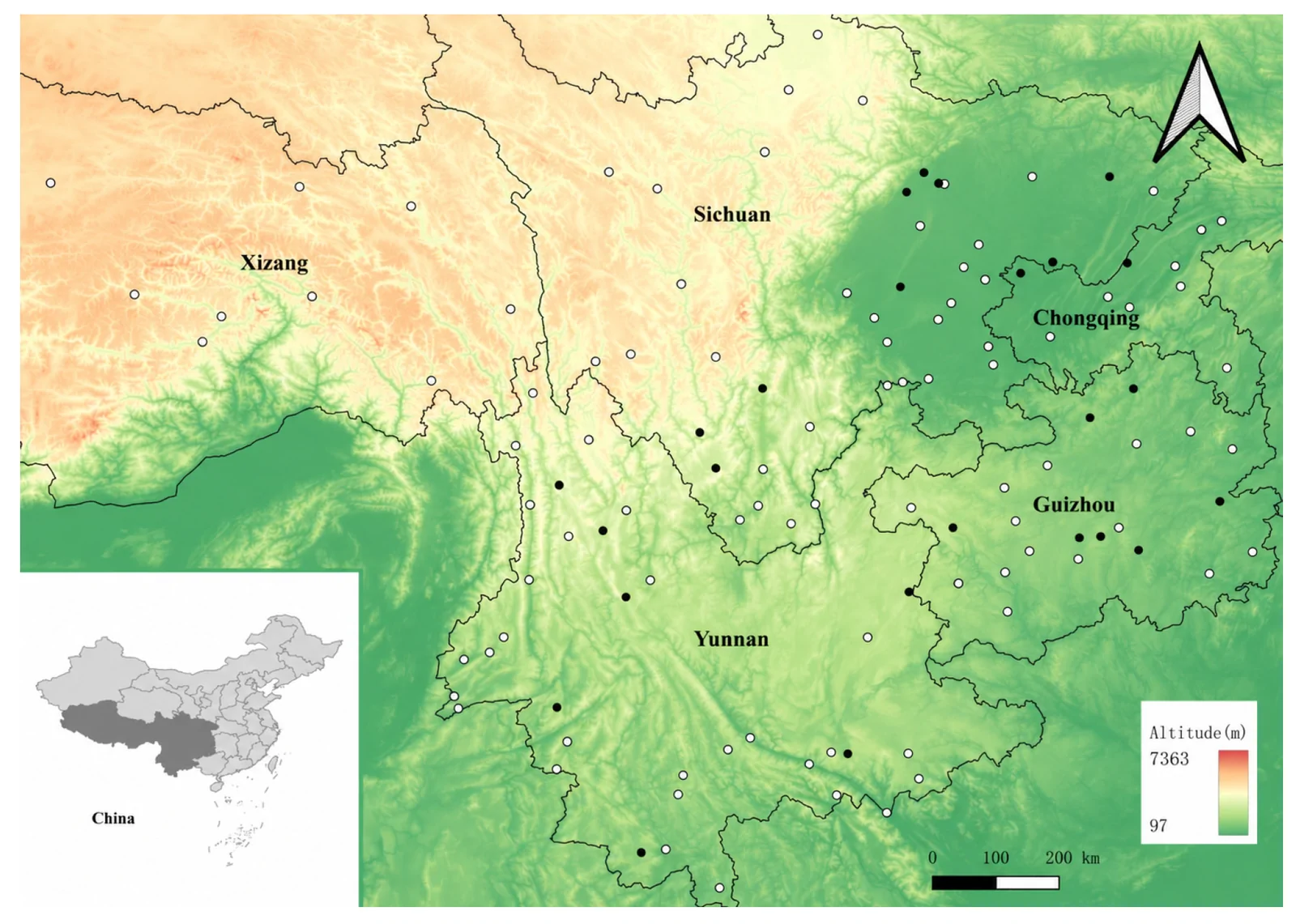
Distribution of survey sites for red-eared harvest mice (*Micromys erythrotis*) in southwest China (2000–2024). Annotation: The hollow circles (○) represent all the survey sites, and the solid circles (●) represent the positive survey sites where *M. erythrotis* were captured.

**Figure 2:**
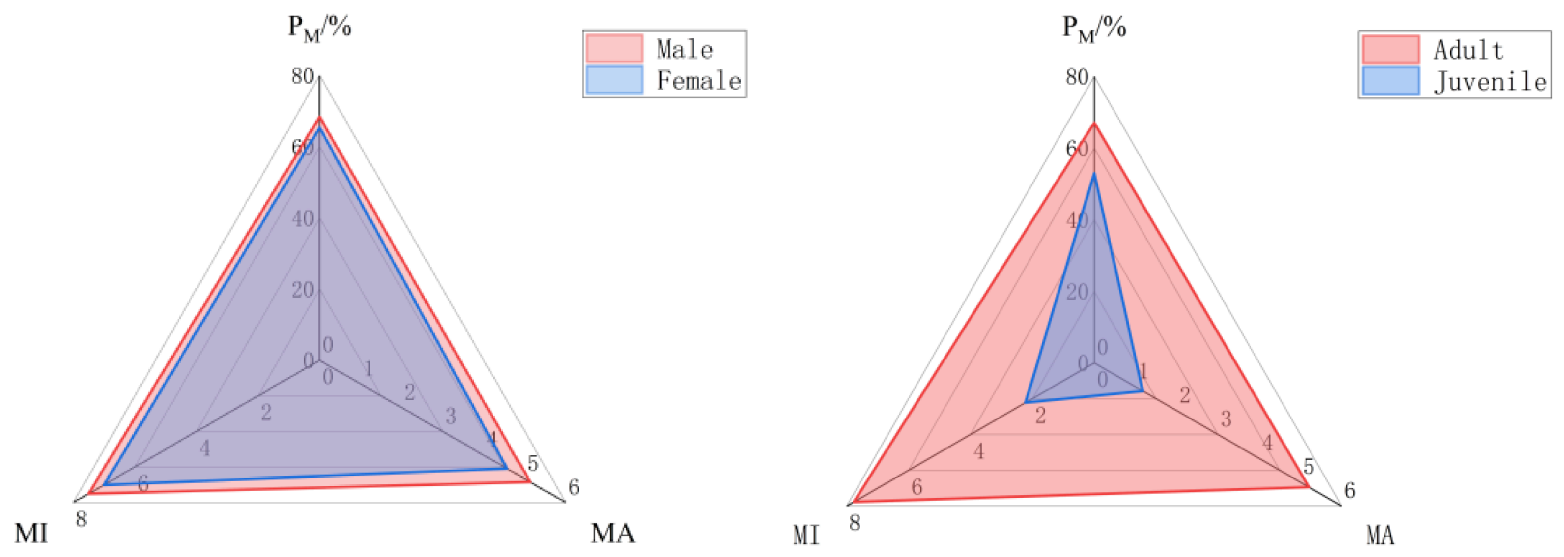
Infestation indices of ectoparasites on different sexes and ages of *Micromys erythrotis* in southwest China (2000–2024).

**Figure 3:**
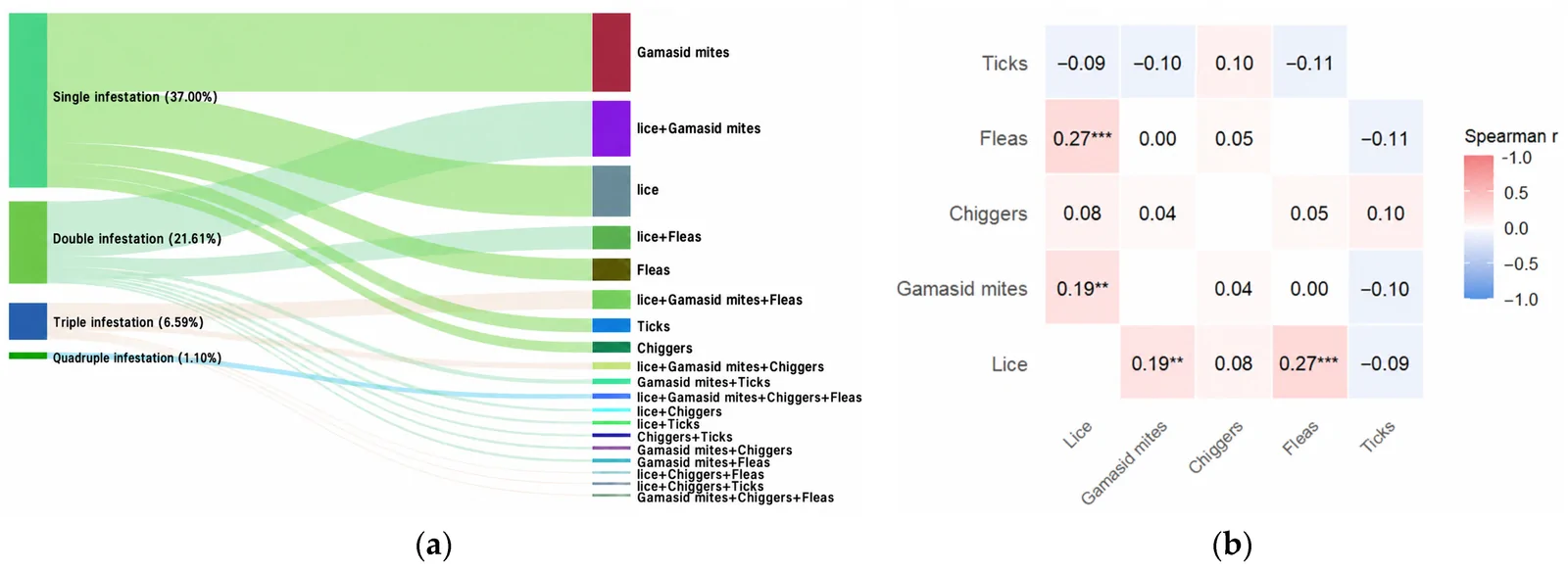
Visualization diagrams for single-group infestation and co-infestations among different parasite groups, and their mutual relationships on *Micromys erythrotis* in southwest China (2000–2024). (**a**) Sankey diagram visualizing the proportions (%) of hosts with single-group infestation and co-infestations among different parasite groups, fleas, sucking lice, gamasid mites, chiggers, and ticks. (**b**) Correlation heatmap visualizing mutual relationships among different parasite groups based on Spearman’s rank correlation, in which the colors indicate the direction and degree of correlations, and the numbers within square cells represent the values of correlation coefficient (*ρ*). Asterisks indicate statistical significance: ** *p* < 0.01 and *** *p* < 0.001.

**Table 2:** Infestation and spatial distribution indices of five ectoparasite groups on *Micromys erythrotis* in southwest China (2000–2024).

Ectoparasite Group	Species and Individual Counts		Infestation Metrics			Spatial Distribution Indices			
	No. of Species	No. of Individuals	Prevalence (*P_M_*, %)	Mean Abundance (*MA*)	Mean Intensity (*MI*)	*C*	*I*	*C_A_*	m*/*m*
Fleas	7	82	16.12	0.30	1.86	2.02	1.02	3.41	4.41
Sucking lice	2	615	36.26	2.25	6.21	12.08	11.08	4.92	5.92
Gamasid mites	24	393	38.10	1.44	3.78	10.91	9.91	6.89	7.89
Chiggers	17	232	8.06	0.85	10.55	27.18	26.18	30.81	31.81
Ticks	4	27	5.86	0.10	1.69	2.39	1.39	14.07	15.07
Total	54	1349	66.30	4.94	7.45	13.75	12.75	2.58	3.58

**Table 3:** Effects of host and environmental factors on ectoparasite infestation on *Micromys erythrotis* (Results of GLMM analysis) in southwest China (2000–2024).

Host and Environmental Factors	Prevalence Model				Parasite Count Model			
	Wald *χ^2^*	OR	95% CI	*p*	Wald *χ^2^*	IRR	95% CI	*p*
Host sex	0.6419	1.317	0.672–2.580	0.423	1.5729	1.289	0.867–1.918	0.210
Host age	0.6204	2.062	0.341–12.486	0.431	0.2569	1.430	0.359–5.705	0.612
Host body weight	0.0739	1.076	0.636–1.819	0.786	0.6349	0.877	0.635–1.211	0.426
Host body length	0.4793	0.824	0.477–1.425	0.489	1.1319	1.172	0.875–1.570	0.287
Habitat type	1.0060	1.663	0.616–4.490	0.316	0.0010	0.990	0.548–1.791	0.974
Landscape type	0.2751	1.592	0.280–9.048	0.600	0.0269	0.894	0.235–3.400	0.870
Elevation	1.2106	1000–2000 m: 0.863 >2000 m: 2.858	0.136–5.487 0.218–37.400	0.5459	1.3641	1000–2000 m: 0.477 >2000 m: 0.806	0.124–1.835 0.137–4.744	0.5056

Note: The random-intercept variance of sampling site was 1.727 for the prevalence model and 1.038 for the parasite count model.

## Data Availability

The data presented in this study are available on request from the corresponding author. The data are not publicly available due to privacy restrictions.

## References

[1] Meerburg B.G., Singleton G.R., Kijlstra A. (2009). Rodent-borne diseases and their risks for public health. Crit. Rev. Microbiol..

[2] Gong L., Diao L., Lv T., Liu Y., Liu J., Zhang W., Xie X., Cao Y. (2025). A comprehensive review of tick-borne disease epidemiology, clinical manifestations, pathogenesis, and prevention. Anim. Zoonoses.

[3] Li W., Li G.C., Liu X.B., Liu Q.Y., Lu L. (2020). Research progress in epidemiological characteristics and influencing factors of scrub typhus. Chin. J. Vector Biol. Control.

[4] Rajamannar V., Govindarajan R., Kumar A., Samuel P.P. (2022). A review of public health important fleas (Insecta, Siphonaptera) and flea-borne diseases in India. J. Vector Borne Dis..

[5] Chen Z., Pei X., Song J., Song W., Shi Z., Onditi K.O., Li Q., Jiang X. (2023). Systematics and evolutionary history of the genus *Micromys* (Mammalia: Rodentia: Muridae). Mamm. Biol..

[6] Yin P.W., Guo X.G., Song W.Y., Dong W.G., Lv Y., Jin D.C. (2024). The on-Site Monitoring and Specimen-Making of Ectoparasites on Rodents and Other Small Mammals. Bio-Protocol.

[7] Huang W.J., Chen Y.X., Wen Y.X. (1995). Rodents of China.

[8] Wei F.W. (2022). Taxonomy and Distribution of Mammals in China.

[9] Wu H.Y., Liu Q., Gong Z.D. (2007). Fauna Sinica: Insecta, Siphonaptera.

[10] Jin D.X. (1999). Classification and Retrieval of Sucking Lice in China.

[11] Deng G.F., Wang D.Q., Gu Y.M., Meng Y.C. (1993). Economic Insect Fauna of China, Fasc. 40, Acari: Dermanyssoidea.

[12] Li J.C., Wang D.Q., Chen X.B. (1997). Trombiculid Mites of China: Studies on Vector and Pathogen of Tsutsugamushi Disease.

[13] Chen Z., Yang X.J. (2021). Systematic Taxonomy of Ticks.

[14] Ding F., Guo X.G., Song W.Y., Fan R., Zhao C.F., Mao K.Y., Zhang Z.W., Peng P.Y., Lin H., Dong W.G. (2021). Infestation and distribution of chigger mites on Brown rat (*Rattus norvegicus*) in Yunnan Province, Southwest China. Trop. Biomed..

[15] Gouma E., Simos Y., Verginadis I., Lykoudis E., Evangelou A., Karkabounas S. (2012). A simple procedure for estimation of total body surface area and determination of a new value of Meeh’s constant in rats. Lab. Anim..

[16] Cheung M.C., Spalding P.B., Gutierrez J.C., Balkan W., Namias N., Koniaris L.G., Zimmers T.A. (2009). Body surface area prediction in normal, hypermuscular, and obese mice. J. Surg. Res..

[17] Benedek A.M., Boeraș I., Lazăr A., Sandu A., Cocîrlea M.D., Stănciugelu M., Cic N.V., Postolache C. (2024). Effects of season, habitat, and host characteristics on ectoparasites of wild rodents in a mosaic rural landscape. Animals.

[18] Liu Z., Guo X.G., Yang Z.H. (2018). Status of research on the flea *Neopsylla specialis*. Chin. J. Pathog. Biol..

[19] Wang L., He Y.M., Ji H.Q., Li Z.F., Zhao T., Huang W., Xiao H.S. (2023). An analysis of surveillance results of small mammals and body surface fleas in the Chongqing section of the Three Gorges Reservoir Region, China, 2019–2021. Chin. J. Vector Biol. Control.

[20] Chen B., Liu Y.-F., Lu X.-Y., Jiang D.-D., Wang X., Zhang Q.-F., Yang G.-P., Yang X. (2023). Complete mitochondrial genome of *Ctenophthalmus quadratus* and *Stenischia humilis* in China provides insights into fleas phylogeny. Front. Vet. Sci..

[21] Zhu X.J., Li Y.N., Guo X.G., Ren T.G. (2026). Distribution and Infestation of Gamasid Mite *Androlaelaps fahrenholzi* (*Haemolaelaps glasgowi*) on Small Mammals Across Five Provincial Regions of Southwest China. Vet. Med. Sci..

[22] Wu X.-B., Na R.-H., Wei S.-S., Zhu J.-S., Peng H.-J. (2013). Distribution of tick-borne diseases in China. Parasites Vectors.

[23] Li L.-H., Zhu D., Zhang C.-C., Zhang Y., Zhou X.-N. (2016). Experimental transmission of *Babesia microti* by *Rhipicephalus haemaphysaloides*. Parasites Vectors.

[24] Zhang S., Gao Z., Wang H., Gao J., Guo F., Wang R., Xing W., Liu J., Zhang X., Yang X. (2025). Acaricidal bioactivity and molecular target analysis of *Origanum onites* and *Ocimum gratissimum* essential oils against *Haemaphysalis doenitzi* ticks. Parasites Vectors.

[25] Li Y.N., Zhu X.J., Ren T.G., Jing Y.G., Yin P.W., Lv Y., Zhang L., Peng P.Y., Guo X.G. (2026). Distribution and infestation of gamasid mite *Laelaps echidninus* (Mesostigmata: Laelapidae) on small mammals across five provincial regions of southwest China. Persian J. Acarol..

[26] Lv Y., Yin P.W., Guo X.G., Fan R., Zhao C.F., Zhang Z.W., Zhao Y.F., Zhang L. (2026). Infestation, Community Structure, and Seasonal Dynamics of Chiggers on Small Mammals at a Focus of Scrub Typhus in Northern Yunnan, Southwest China. Insects.

[27] Zhu X.-J., Li Y.-N., Guo X.-G., Ren T.-G., Jing Y.-G., Zhang L., Qian T.-J. (2026). Infestation patterns and ecological distribution of fleas and sucking lice on *Rattus tanezumi* in southwest China: Evidence from a long-term multi-provincial study (2000–2024). Vet. World.

[28] Krasnov B.R., Bordes F., Khokhlova I.S., Morand S. (2012). Gender-biased parasitism in small mammals: Patterns, mechanisms, consequences. Mammalia.

[29] Shilereyo M., Magige F., Ranke P., Ogutu J., Røskaft E. (2022). Ectoparasite load of small mammals in the Serengeti Ecosystem: Effects of land use, season, host species, age, sex and breeding status. Parasitol. Res..

[30] Krasnov B.R., Matthee S., Lareschi M., Korallo-Vinarskaya N.P., Vinarski M.V. (2010). Co-occurrence of ectoparasites on rodent hosts: Null model analyses of data from three continents. Oikos.

